# Transforaminal endoscopic lumbar foraminotomy for octogenarian patients

**DOI:** 10.3389/fsurg.2024.1324843

**Published:** 2024-02-01

**Authors:** Yong Ahn, Sung-Kyu Song

**Affiliations:** Department of Neurosurgery, Gil Medical Center, Gachon University College of Medicine, Incheon, Republic of Korea

**Keywords:** endoscopic procedures, lumbar foraminal stenosis, octogenarians, radiculopathy, transforaminal endoscopic lumbar foraminotomy

## Abstract

**Background:**

Radiculopathy caused by lumbar foraminal stenosis in older people has become more common in the aging general population. However, patients aged ≥80 years rarely undergo conventional open surgery under general anesthesia because of the high risk of peri-operative morbidity and adverse events. Therefore, less invasive surgical alternatives are needed for older or medically handicapped patients. Transforaminal endoscopic lumbar foraminotomy (TELF) under local anesthesia may be helpful in at-risk patients, although only limited information is available regarding the clinical outcomes of this procedure in octogenarians. Therefore, this study aimed to investigate the safety and efficacy of TELF for treating radiculopathy induced by foraminal stenosis in octogenarian patients.

**Methods:**

Overall, 32 consecutive octogenarian patients with lumbar foraminal stenosis underwent TELF between January 2019 and January 2021. The inclusion criterion was unilateral radiculopathy secondary to lumbar foraminal stenosis. The pain focus was confirmed using imaging studies and selective nerve blocks. Full-scale foraminal decompression was performed using a percutaneous transforaminal endoscopic approach under local anesthesia. Surgical outcomes were assessed using the visual analog pain score, Oswestry Disability Index, and modified MacNab criteria.

**Results:**

The pain scores and functional outcomes improved significantly during the 24-month follow-up period, and the rate of clinical improvement was 93.75% in 30 of the 32 patients. None of the patients experienced systemic complications.

**Conclusion:**

TELF under local anesthesia is an effective and safe treatment for foraminal stenosis in octogenarian or medically compromised patients. The mid-term follow-up did not reveal any significant progression in spinal stability. Therefore, this endoscopic procedure can be an effective alternative to aggressive surgery for managing lumbar foraminal stenosis in octogenarian patients with intractable radiculopathy.

## 1 Introduction

Modern society is characterized by an increasingly aging population and complex lifestyle. Therefore, quality of life is of paramount importance. The invasiveness and efficiency of treatments have become primary issues in patients with degenerative spinal diseases.

Despite conservative treatment, lumbar foraminal stenosis frequently results in severe radicular pain. Extensive open foraminal decompression, with or without fusion, may be required for intractable lumbar foraminal stenosis. However, the irritation of the dorsal root ganglion and corner-side neural pinches may result in surgical morbidity and postoperative flares. Particularly, older surgical candidates with lumbar foraminal stenosis are at high risk of broad surgical exposure under general anesthesia. Additionally, the surgical requirements for older patients with lumbar foraminal stenosis increase as the longevity of the population increases.

Several studies have reported higher peri-operative complication rates in geriatric patients (1–5). Particularly, patients aged ≥75 years who underwent spinal surgery showed a significantly higher rate of major complications (2, 6). Some authors have reported considerable risks of general anesthesia, such as cardiovascular morbidity, pulmonary dysfunction, and impaired cognitive function, in older patients (7–11). Regarding spine disorders, surgeries in patients aged >80 years have higher medical risks and longer lengths of hospital stay than those in younger patients (12–15). However, some authors have reported that spine surgery for octogenarian patients is worthwhile with acceptable surgical complication rates (16–19). Therefore, a less invasive and safer alternative surgical technique is required for octogenarian patients with lumbar degenerative stenosis.

Transforaminal endoscopic lumbar foraminotomy (TELF) is a minimally invasive surgical alternative to treat symptomatic lumbar foraminal stenosis (20–25). Under local anesthesia, it involves full-scale foraminal decompression through a percutaneous tissue-preserving transforaminal approach. Percutaneous endoscopic lumbar foraminoplasty or foraminotomy was first performed in the late 1990s. Knight et al. (20, 26) introduced an endoscopic lumbar foraminal decompression technique using a side-firing laser. Some authors have reported endoscopic foraminal decompression using bone trephines (22, 27). As endoscopic technology has evolved, modern endoscopic foraminal decompression has enabled full-scale foraminal decompression using various specialized surgical tools, such as endoscopic burrs, steerable forceps, and micropunches (24, 28). Notably, the current TELF technique can treat most types of lumbar foraminal stenosis, from mild dynamic foraminal narrowing to severe foraminal stenosis with collapsed disc space or spondylolisthesis (29, 30). Therefore, this technique may be helpful for older or medically-compromised patients at risk for extensive surgery under general anesthesia. Although many authors have published case series or cohort studies on TELF or similar techniques, clinical studies on TELF in older patients (≥80 years) are lacking.

Therefore, this study aimed to demonstrate the surgical outcomes of TELF in octogenarian patients with lumbar foraminal stenosis without segmental instability and discuss the advantages and disadvantages of this minimally invasive procedure.

## 2 Materials and methods

### 2.1 Patients

Patient data were prospectively entered into the database, and the records were retrospectively reviewed. Retrospective data were collected from 32 consecutive patients aged ≥80 years who underwent TELF between January 2019 and January 2021. This study was approved by the institutional review board, and written informed consent was obtained from all patients. The eligibility criteria for this study were as follows: (1) patients aged ≥80 years with chronic radicular leg pain, with or without back pain; (2) definitive lumbar foraminal stenosis confirmed by both computed tomography (CT) and magnetic resonance imaging (MRI); (3) foraminal pain source confirmed by selective nerve root block; and (4) failure of extensive conservative treatments for at least 6 weeks. The exclusion criteria included cauda equina syndrome, spondylolisthesis, segmental instability in the sagittal and coronal plane, and coexisting pathological conditions, such as systemic neuropathy, infection, and spinal tumors.

### 2.2 Surgical technique

**Figure 1:**
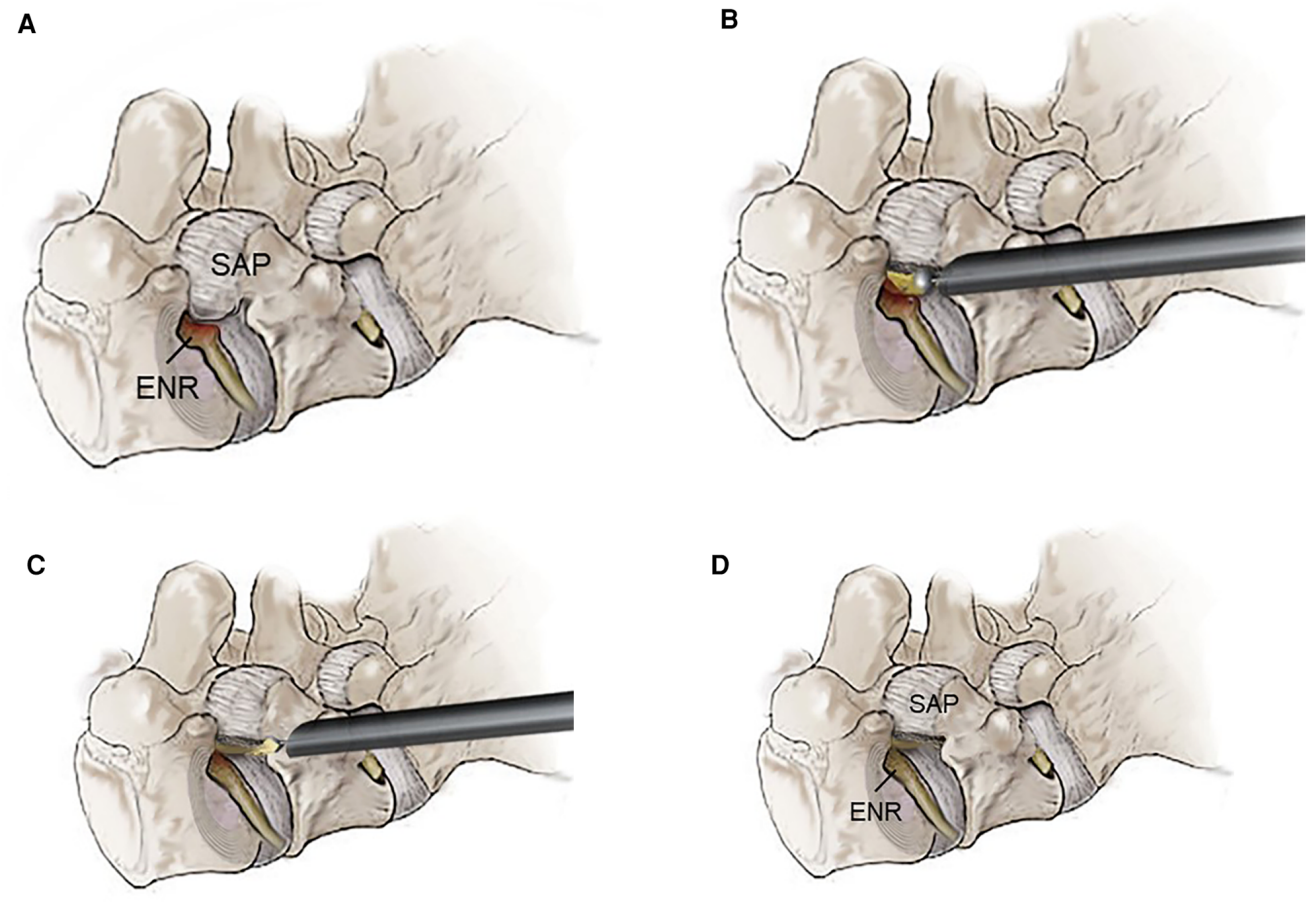
The principal concept of transforaminal endoscopic lumbar foraminotomy. (**A**) Severe foraminal stenosis compresses the exiting nerve root (ENR) due to the hypertrophic superior articular process (SAP) and ligaments. (**B**) Foraminal unroofing using endoscopic burrs and micropunches for resecting the tip of the superior articular process and osteophytes. (**C**) Neural release with removal of the ligamentum flavum, foraminal ligaments, and redundant disc. (**D**) The final point of the full-scale foraminal decompression from the axillary side to the lateral exit zone. Note the resected SAP and decompressed ENR.

The surgical technique was performed in three stages, according to the standard ELF technique (24, 28): (1) extraforaminal docking of the working cannula under fluoroscopic guidance, (2) foraminal unroofing using trephines and burrs, and (3) full-scale foraminal decompression under endoscopic control (Figure 1). Conscious sedation was administered for anesthesia. Before surgery, 0.05 mg/kg of intramuscular midazolam and 0.8 μg/kg of intravenous fentanyl were administered. Additional fentanyl was administered according to the patient's condition and surgeon's needs.

#### 2.2.1 Transforaminal approach (outside-in technique)

**Figure 2:**
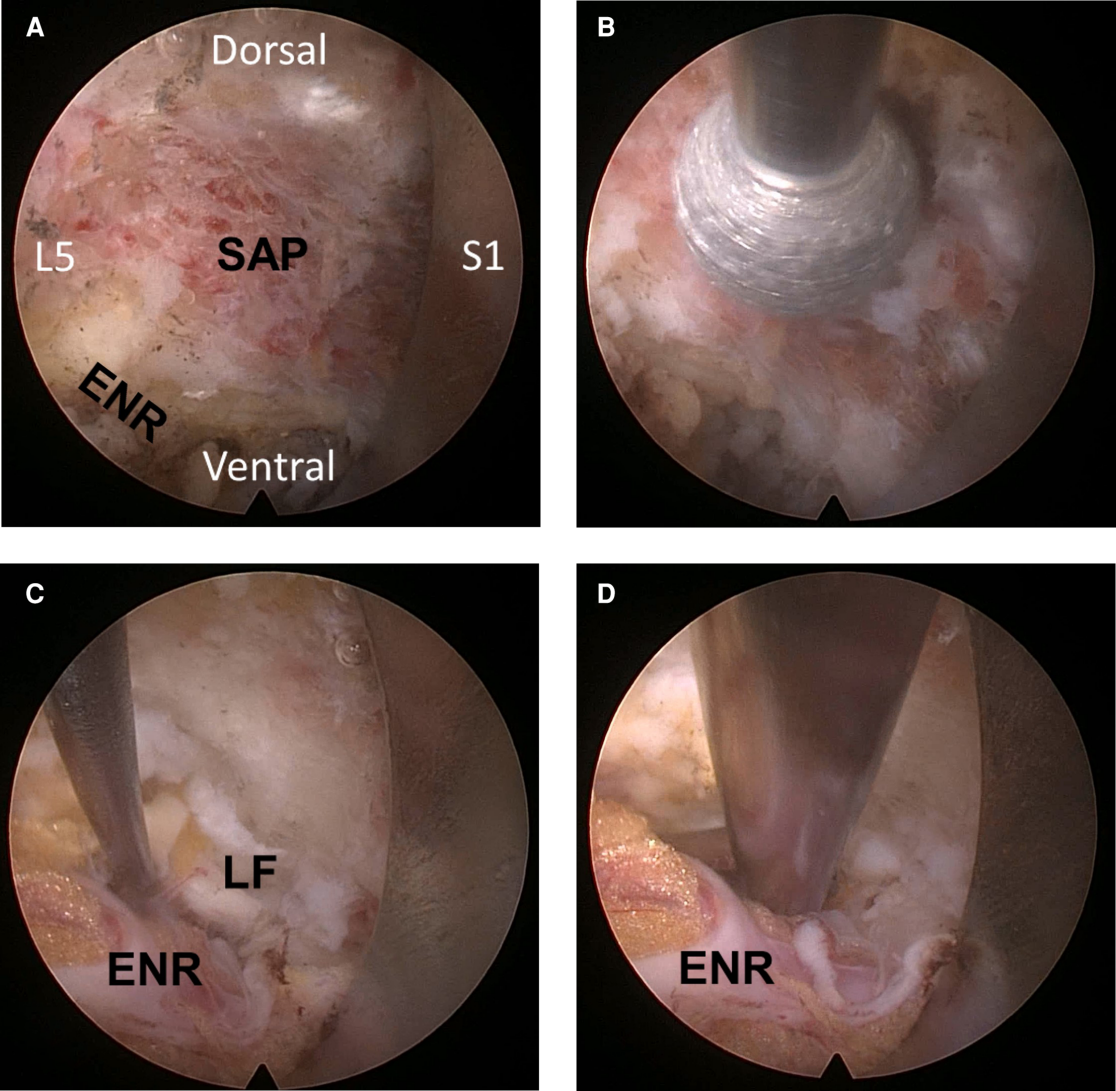
Intra-operative endoscopic pictures. (**A**) The initial operative field includes the disc surface, exiting nerve root (ENR), and the surface of the superior articular process (SAP). (**B**) Foraminal unroofing along the synovial joint, removing the tip of the SAP. (**C**) Soft tissue decompression with the removal of hypertrophic ligamentum flavum to the axillary epidural space. (**D**) The ENR becomes freely released and mobilized through the entire path at the final point.

The first step of this procedure was extraforaminal docking of the working cannula to expose the stenotic foraminal zone and pinched nerve root. An 18-gauge spinal needle was inserted toward the surface of the superior articular process (SAP) and the continuing pedicle. Therefore, the exiting nerve root (ENR) was preserved in the stenotic foramen during the approach under fluoroscopic control. The needle could be firmly engaged in the facet joint and subsequently replaced by a guidewire. A tapered obturator was inserted over the guidewire and advanced into the intervertebral foramen with gentle manual rotation. After correctly placing the obturator in the foramen (rather than in the disc), a bevel-ended working sheath was advanced over the obturator with the sharp end directed opposite the ENR and placed on the undersurface of the facet joint. An ellipsoidal working-channel endoscope was inserted after the obturator was withdrawn. Ideally, the bevel-ended working sheath should be firmly engaged (docked) in the foramen, and the periforaminal anatomy should be viewed, including the SAP, ENR, pedicle, and redundant disc. In summary, the working space was initially created outside the foramen, and the decompression process followed the direction of the intraforaminal space (outside-in approach; Figure 2A).

#### 2.2.2 Endoscopic bone work (foraminal unroofing)

Decompression of the foraminal stenosis and neural adhesion was initiated with foraminal unroofing and widening. First, the hypertrophic SAP was resected using a bone-removing trephine or endoscopic burrs. Trephines can be used for rapid bone resection under fluoroscopic guidance. Recently, an endoscopic burr was shown to safely undercut the bone under endoscopic control. Various types of burrs can be used for foraminal unroofing, such as round diamond, side-cutting, and articulating bone burrs. Particularly, wide bone resection was feasible with the articulating burr. The typical direction of bone removal, while preserving the ENR, should be from caudal to cranial and outside-in for the foraminal portion. The most important landmark was the interface between the inferior articular process and SAP. Subsequently, the entire SAP tip was removed when drilling was performed along the interface (Figure 2B), and foraminal unroofing was performed until the ligamentum flavum and epidural fat appeared. Foraminal structures, such as the foraminal ligament, ligamentum flavum, perineural fat covering the ENR, shoulder osteophytes, and disc surface, were assessed after bony decompression. The sharp end of the bevel-ended working sheath was used as an effective neural retractor by rotating the cannula.

#### 2.2.3 Endoscopic soft tissue work (full-scale foraminal decompression)

**Figure 3:**
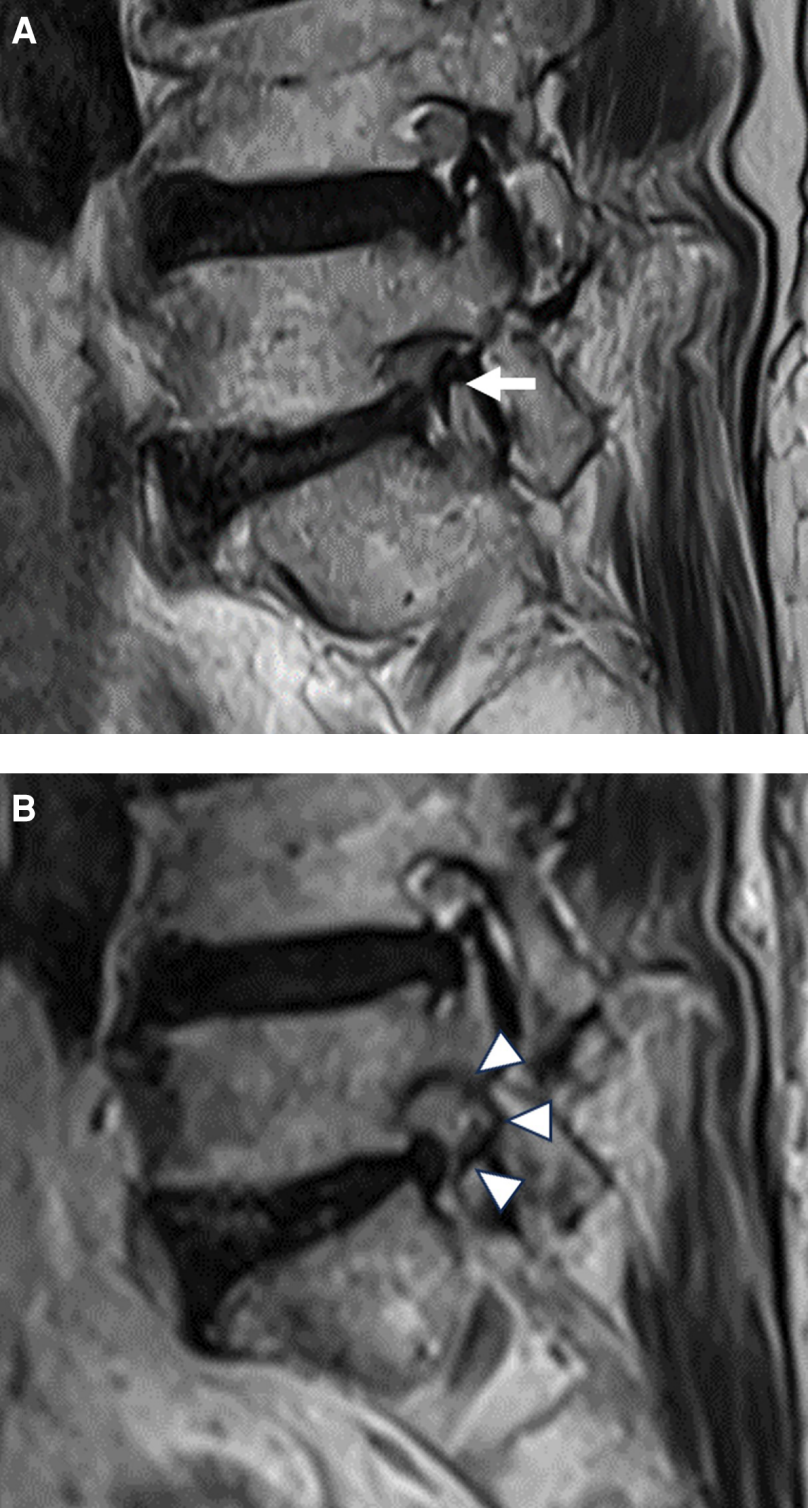
An illustrated case of an 82-year-old female patient. (**A**) Preoperative magnetic resonance image (MRI) showing severe foraminal stenosis at the L5–S1 level (arrow). (**B**) Postoperative MRI showing foraminal decompression with removal of the superior articular process and foraminal ligaments (arrowheads).

After adequate foraminal bone resection and unroofing using endoscopic burrs and punches, the offending soft tissues and adhesions were decompressed using various instruments (Figure 2C). Subsequently, the hypertrophic ligamentum flavum compressing the neural tissues was removed using micropunches and endoscopic punches. The extruded discs, if any, were removed using grasping forceps. Contained redundant discs and soft tissue adhesions were lysed using micropunches and dissecting probes. Other ventral ligaments and shoulder osteophytes were removed using micropunches and semi-flexible forceps. Bipolar radiofrequency was useful for shrinking the disc, dissecting neural adhesions, and controlling epidural bleeding. A flexible probe, under both endoscopic and fluoroscopic guidance, was used to confirm decompression. As the offending adhesion was released, the ENR and dural sac were defined and mobilized. Surgeons were careful to avoid tearing the dural membrane. The endpoint of the procedure was the free mobilization of the ENR and dural sac (Figure 2D). Simple exposure of neural tissues was insufficient. Neural tissues were released from the tough soft-tissue adhesions or anchorages. Next, the endoscope was withdrawn with adequate hemostasis after full-scale foraminal decompression was confirmed. A sterile dressing was applied using a one-point subcutaneous suture. Patients were monitored for at least 6 h for any adverse events. Postoperative MRI or CT was used for precise pathological assessment, as required (Figure 3). Finally, the patient was discharged in the absence of complications within 24 h.

### 2.3 Outcome evaluation and statistical analysis

Follow-up data were obtained using patient-based outcome questionnaires administered during routine outpatient clinic visits or telephone interviews. The pain severity and functional status were assessed using the visual analog scale (VAS) and Oswestry Disability Index (ODI) (31). The global results were classified into four groups using the modified MacNab criteria (32): excellent (no pain and no functional restrictions), good (occasional back/leg pain, brief functional restrictions), fair (improved overall function, permanent work, and activities of daily living modification), and poor (no improvement of pain/function or an index-level reoperation).

The demographics and global outcomes of the octogenarian patient group were compared with those of the younger patient group who underwent TELF under similar surgical indications during the same period.

Statistical analyses were conducted by an independent statistician using SPSS (version 14.0; SPSS Inc., Chicago, Ill., USA). The comparison of pain scores and functional status between pre-operative and postoperative clinical outcomes was performed using repeated measures analysis of variance and paired t-tests with the Bonferroni method for adjusting multiple comparisons. Statistical significance was set at *p* < 0.05.

## 3 Results

This study included 32 patients (18 females and 14 males) with a mean age of 82.97 (range, 80–89) years. The operated levels were L2–3, L3–4, L4–5, and L5–S1 in 1 (3.13%), 6 (18.75%), 12 (37.50%), and 3 (40.63%) patients, respectively. Comorbidities included hypertension (*n* = 16, 50%), diabetes (*n* = 14, 43.75%), coronary heart disease (*n* = 10, 31.25%), Parkinson's disease (*n* = 5, 15.63%), and cognitive issues (*n* = 4, 12.50%). The mean operative duration was 60.56 (range: 33–85) min.

**Figure 4:**
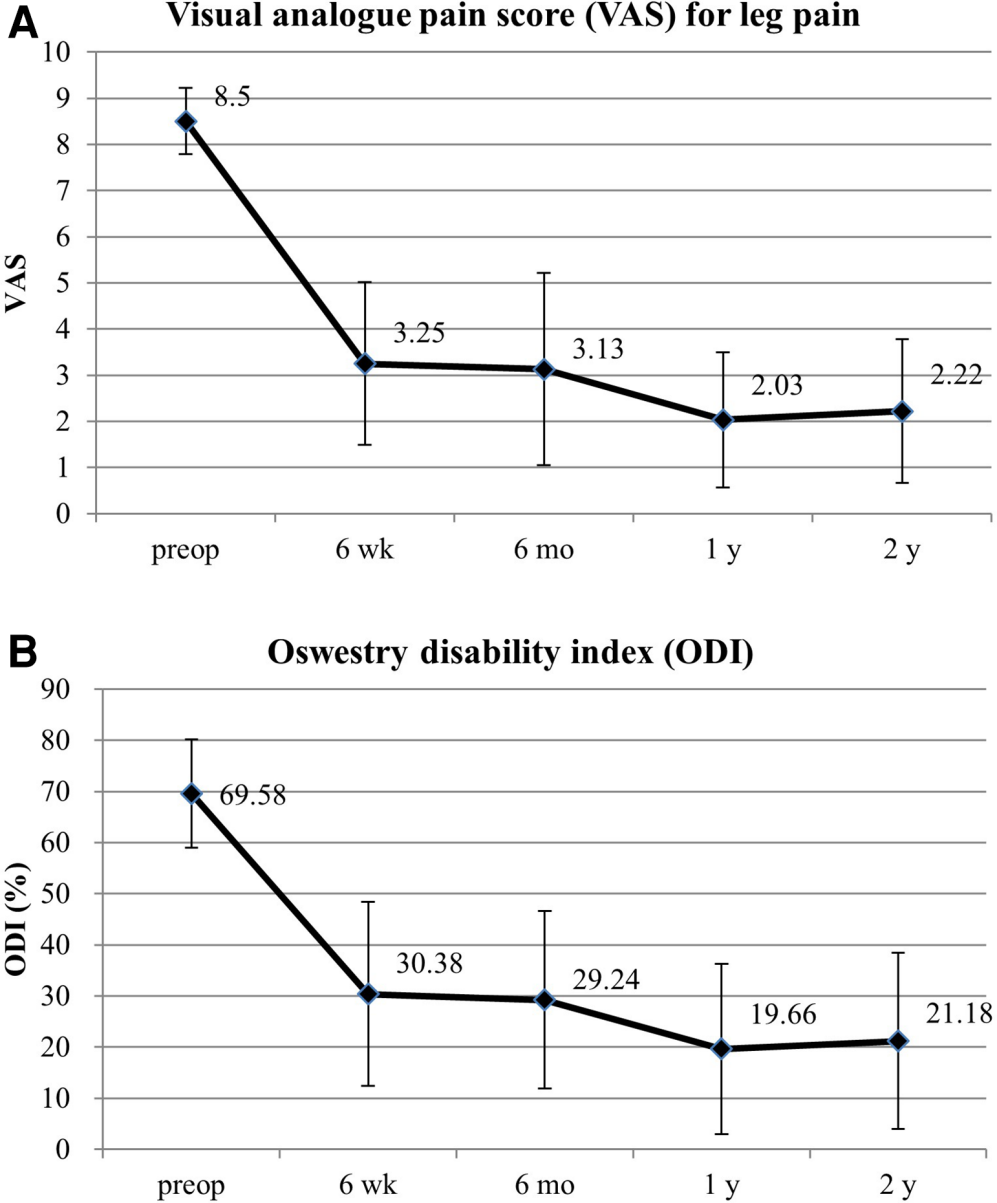
The clinical results. (**A**) Visual analog scores for radicular leg pain pre-operatively and at 6 weeks, 6 months, 1 year, and 2 years postoperatively. (**B**) Oswestry Disability Index scores pre-operatively and at 6 weeks, 6 months, 1 year, and 2 years postoperatively.

The VAS score [mean ± standard deviation (SD)] for the lumbar radiculopathy significantly improved from 8.50 ± 0.72, pre-operatively, to 3.25 ± 1.76, 3.13 ± 2.08, 2.03 ± 1.47, and 2.22 ± 1.56 at 6 weeks, 6 months, 1 year, and 2 years postoperatively, respectively (*p *< 0.001, Figure 4A). Additionally, the ODI score (mean ± SD) improved from 69.58 ± 10.55% pre-operatively to 30.38 ± 17.97%, 29.24 ± 17.38%, 19.66 ± 16.64%, and 21.18 ± 17.25% at 6 weeks, 6 months, 1 year, and 2 years postoperatively, respectively (*p *< 0.001; Figure 4B).

**Figure 5:**
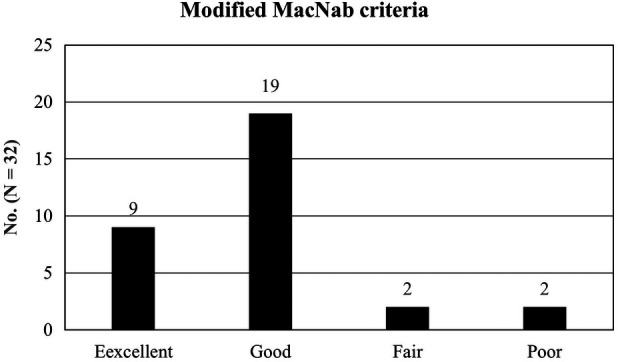
The global outcomes based on the modified macNab criteria: excellent in 7 patients (21. 88%), good in 21 (65.63%), fair in 3 (9.38%), and poor in 1 (3.13%). Therefore, the success rate was 87.50%, and the clinical improvement rate was 96.88%.

The overall clinical outcomes according to the modified MacNab criteria were excellent, good, fair, and poor in 7 (21.88%), 21 (65.63%), 3 (9.38%), and 1 (3.13%) patients, respectively. Therefore, the rate of symptomatic improvement was 96.88% (Figure 5). Among the 32 patients, 1 with poor outcomes experienced sustained radicular pain and postoperative flares. The patients were treated with repeated postoperative nerve blocks and oral medications. One patient experienced a minor dural tear and was treated with sealing materials intra-operatively. Furthermore, no other significant postoperative complications occurred, and no changes in segmental stability were documented in radiological studies during the follow-up period.

Operative data and the global outcomes were compared with patients aged <80 years who underwent TELF during the same period. The success rate based on the modified Macnab criteria and complication rate did not differ between the two age groups (Table 1).

**Table 1:** Comparison between octogenarian and younger patients.

	Octogenarian (*n* = 32)	Younger (*n* = 97)	*p*-values
Age (mean, y)	82.97 (80–89)	66.23 (42–79)	<0.0001
Male:Female	14:18	48:49	NS
Operative level			NS
L2–3	1	1	
L3–4	6	9	
L4–5	12	40	
L5–S1	13	47	
MacNab criteria			NS
Excellent	7 (21.88%)	25 (25.77%)	
Good	21 (65.63%)	62 (63.92%)	
Fair	3 (9.38%)	7 (7.22%)	
Poor	1 (3.13%)	3 (3.09%)	
Reoperation (%)	0	1 (open foraminotomy)	NS
Complication (%)			NS
Infection	0	0	
Hematoma	0	1 (minor, muscle)	
Dural tear	1 (minor, intraoperative)	0	
Dysesthesia	2 (1 transient, 1 persistent)	5 (transient)	

NS, not significant.

## 4 Discussion

### 4.1 Data interpretation

Our results revealed that the clinical efficacy was significant for pain scores, disability indices, and global functional status. The mean VAS score for radicular pain improved by 6.28 at the final interview (*p* < 0.001), and the mean ODI improved by 48.40 at the final follow-up (*p* < 0.001). Generally, a change of >50% in the VAS score (33) and a reduction of >30% in the ODI (34, 35) are clinically relevant. According to published data on the outcomes of TELF for all age groups (24), the mean reduction in the VAS score and the mean improvement in the ODI were 6.36 and 46.5, respectively. Therefore, TELF for octogenarian patients resulted in relevant and comparable outcomes. Pain and functional status steadily improved until 1-year postoperatively and subsequently slightly worsened or stabilized, which may be related to the natural course of lumbar degeneration in older patients.

The global outcomes, including the modified McNab criteria, revision surgery, and complications, in older patients were also similar to those of younger patients who underwent TELF during the same period (Table 1).

Most older patients experience concurrent medical issues and risks associated with general anesthesia. TELF under local anesthesia can prevent the systemic adverse effects of open surgery under general anesthesia in addition to its clinical relevance.

### 4.2 Benefits and cons of the endoscopic procedure

TELF under local anesthesia can provide sufficient decompression of foraminal stenosis in older patients. Endoscopic procedures have several benefits. First, the endoscopic technique is adequate for foraminal decompression because the foraminal or extraforaminal zone is narrow and may result in unbearable radiculopathy. Therefore, a small amount of tissue removal and adhesion release can relieve severe pain. Second, a percutaneous approach with a minimal stab incision may reduce musculoskeletal tissue damage and the risk of complications, including muscle atrophy, segmental instability, surgical site infection, or hematoma. Third, the short operative time under local anesthesia may facilitate postoperative recovery without the risk of general anesthesia in geriatric patients. Finally, the endoscopic procedure, which is known as a “minimally invasive” technique, is not minimally effective. Previously, endoscopic spine surgery was criticized as a gimmick or transiently effective procedure. However, the modern concept of transforaminal endoscopic spine surgery has proven to be as effective as standard open surgery (36–38). Therefore, the transforaminal endoscopic surgical approach is appropriate for older patients with medical issues as well as younger patients.

In contrast, the long learning curve and technical difficulties may be critical barriers to entry for this minimally invasive technique (39). Surgeons can obtain relevant and reproducible outcomes only after reaching a significant proficiency level. Therefore, the clinical applications of TELF should be carefully considered.

### 4.3 Technical keys

For elderly and medically compromised patients, the procedure should be performed as quickly as possible under adequate anesthesia while maintaining a safe range of vital signs. Therefore, an efficient and standardized endoscopic foraminal decompression technique is required. First, the ENR irritation should be prevented during the transforaminal approach. Mechanical damage to inflamed ENR may cause significant postoperative dysesthesia and lead to poor clinical outcomes. The initial landing point should be as close as possible to the ENR to prevent nerve root irritation. Based on our experience, the posterior vertebral body surface near the upper endplate of the disc is recommended. The sharp end of the working sheath should be directed away from the ENR to allow the surgeon to view the epidural fat in the initial endoscopic visual field. Immediate and extensive conservative treatments, including a selective nerve root block with specific medications for neuropathic pain, are required in the case of postoperative dysesthesia. Second, foraminal decompression should be directed obliquely to the axillary point along the ENR and not horizontally parallel to the disc space. The direction landmark of SAP resection is the synovial joint between the pedicle and SAP tip. Removal of the SAP tip and hypertrophic ligaments along the synovial joint is the key to efficiently decompress the pinched ENR. In contrast, decompression parallel to the disc space is ineffective and subject to incomplete decompression of the remaining parts of the bone and ligaments. Finally, the surgeon must confirm the axillary epidural space and free mobilization of the ENR to complete the procedure. Exposure of the ENR alone is insufficient for full-scale foraminal decompression. We can observe the ENR during the process, even at an early stage. However, surgeons must continue to decompress the exposed ENR until the neural tissue is released. Once released, the ENR begins to beat according to the arterial beat and epidural pressure. Therefore, a sufficient margin of decompression around the nerve root should be made to prevent recurrent stenosis caused by segmental instability or degenerative change over time.

### 4.4 Limitations and future perspective

Our study had some limitations. First, the analysis was performed retrospectively without an adequate control group. Therefore, selection bias may have been involved in patient inclusion. Secondly, the number of patients was relatively small to draw reliable conclusions. Finally, the generalizability of this study was limited by the fact that the endoscopic procedures were performed by a single surgeon at a single institution. Therefore, a long-term prospective cohort study or randomized trial comparing the outcomes between the TELF and conventional open surgery groups with a more significant number of older patients is required to verify the endoscopic procedure's effectiveness. However, this study suggests that a percutaneous endoscopic procedure is feasible in older patients with various foraminal stenoses, preventing general anesthesia and extensive open surgery.

As more people live longer, the number of octogenarians or older patients with degenerative spine disease is predicted to increase. Therefore, the need for endoscopic procedures performed under local anesthesia is increasing. Our data revealed that using TELF for lumbar foraminal stenosis resulted in relevant and reliable clinical outcomes. However, it requires a steep learning curve and sufficient experience for aspiring endoscopic spine surgeons to achieve relevant outcomes with this minimally invasive endoscopic technique. Surgical approaches, devices, and optical technologies have advanced remarkably. Ultimately, the conservative spine society should accept this kind of novel endoscopic technique as a mainstream of spine surgery, according to people's needs.

## 5 Conclusion

Octogenarian patients with symptomatic radiculopathy caused by lumbar foraminal stenosis have a considerable risk of developing perioperative morbidities. Full-scale foraminal decompression via a transforaminal endoscopic approach under local anesthesia is feasible and safe for older or medically compromised patients. Additionally, specialized endoscopic techniques are essential for clinical success. Therefore, enthusiasts of endoscopic spine surgery should consider increasing their skills and experience to achieve relevant clinical outcomes.

## Data Availability

The raw data supporting the conclusions of this article will be made available by the authors, without undue reservation.
